# Exploring Medical Students’ Perceptions of the Clinical Priorities of Eating Disorders

**DOI:** 10.1192/bjo.2026.11331

**Published:** 2026-06-30

**Authors:** Ella Chamberlain, David Coyle, Emanuele Fino

**Affiliations:** 1 Queens University Belfast, Belfast, United Kingdom; 2 Belfast Health and Social Care Trust, Belfast, United Kingdom

## Abstract

**Aims:**

The presence of weight bias in clinical professionals’ perceptions of eating disorders has been highlighted in previous research. This can cause patients to feel dismissed, leading to a poor clinician–patient relationship, and can delay referral for patients in need of urgent treatment.

This study will explore how medical students perceive the severity and therefore priority for referral to secondary care of various eating disorder presentations, in order to assess how early into a medical career this weight bias is learned. This will be done through a survey asking participants to rank 8 fictional patient cases in the order of priority for referral that they deem most appropriate. The cases range in BMI, with some having a lower BMI with less clinically urgent signs, and some having a higher BMI with more clinically urgent signs.

The aim is to determine the extent of weight bias in the medical student population by comparing their rankings to an eating disorder consultant’s ranking which will highlight any tendencies of participants to rank patients with a lower BMI as higher priority regardless of other clinical signs. There are also demographic questions in order to evaluate how gender and year of study impact perceptions. The results will be presented to the medical school to inform future teaching practices and therefore improve future patient care.

**Methods:**

The study will use purposive sampling of Queen's University Belfast medical students from years 1 to 5. The target sample size is around 50, based on a one-sample t-testdesign. Exclusion criteria are as follows; under the age of 18, personal experience of an eating disorder. The latter is to protect potentially vulnerable students, and to ensure that the data is based solely on medical school teaching practices.

Analysis will be done via a t-test, to evaluate any significant differences between the students’ and the consultant’s rankings, and between different genders and year groups. There will also be qualitative interpretation of any justifications or comments left in the free text question.

**Results:**

The study is in progress so there are currently no results, but it is expected to have finished by the International Congress therefore results should be available to present.

**Conclusion:**

In conclusion, weight bias in eating disorder care has a significant impact on patient satisfaction and safety, and undergraduate training provides an opportunity to reduce the stigma that future doctors carry with them into practice. This study aims to improve that training.

